# REMS Logic Model: A Pragmatic Framework for Incorporating Drug Safety into Clinical Practice

**DOI:** 10.1007/s40264-025-01542-9

**Published:** 2025-04-09

**Authors:** Gita A. Toyserkani, Suzanne B. Robottom, Elaine H. Morrato

**Affiliations:** 1 Center for Drug Evaluation and Research, United States Food and Drug Administration, Silver Spring, MD USA; 2 Parkinson School of Health Sciences and Public Health, Loyola University Chicago, Chicago, IL USA; 3 Institute for Translational Medicine, Loyola University Chicago, Chicago, IL USA

More than 163 million Americans—or half of the nation—used at least one prescription medicine in the past 30 days, with nearly one in eight taking five or more medicines [1]. In the USA, nearly three-quarters of outpatient visits involve drug therapy, resulting in 1 billion prescriptions written annually [1]. The US Food and Drug Administration (FDA) is the federal agency responsible for protecting the public’s health by assuring the safety and efficacy of drug and biological products. The safety of prescription drugs is important. Unfortunately, adverse drug events are a significant cause of morbidity in the USA, leading to excess emergency department visits and hospitalizations, especially for people aged ≥ 65 years [2]. For certain drugs that pose a serious risk, the FDA may determine that risk evaluation and mitigation strategies (REMS) are required to ensure that the benefits of a drug outweigh its risks [3].

REMS programs are FDA-mandated public health interventions implemented at a national scale in healthcare settings to achieve an intended drug safety goal. As part of a REMS program, the FDA may require a drug manufacturer to distribute a medication guide for patients and/or implement a communication plan to inform health professionals about a risk and associated safe-use behaviors. For drugs that pose a serious risk of abuse or overdose, the FDA may require the drug manufacturer to develop certain packaging or a safe disposal system. It may also require a drug manufacturer to implement elements to assure safe use, such as specialized training or certification of healthcare providers who prescribe and pharmacists and others who dispense the drug, and/or restricting distribution and dispensing to only certain healthcare settings or with evidence of safe-use conditions, such as laboratory test results. Further, each patient using a drug with a REMS requirement may also be subject to monitoring or enrolled in a registry. As reported on the FDA’s REMS Public Dashboard, there are 73 active REMS as of January 15, 2025, with 69 of the active REMS (94.5% of programs) requiring elements to assure safe use [4]. Examples of drugs with REMS include isotretinoin, opioid analgesics, certain psychotropic medications such as esketamine and long-acting injectable olanzapine, and certain oncology products such as thalidomide and two of its analogues and chimeric antigen receptor T-cell therapies. The FDA maintains a dedicated public website (“REMS@FDA”) that provides an informational database on all currently approved REMS, and information on historical and released REMS is also available [5].

The FDA has worked to increase transparency to help healthcare professionals and the public understand the FDA’s processes and decisions related to REMS and to promote REMS innovation through a variety of mechanisms, including by issuing guidance to industry on developing, implementing, and assessing REMS, holding public workshops, and developing publicly available datasets, such as those found at REMS@FDA and through the Public REMS Dashboard initiative [6].

However, these efforts have not fully addressed the challenges with REMS. Concerns have been raised about the design of REMS programs themselves and the administrative burdens placed on already stretched healthcare professionals, patients, and healthcare systems [7, 8]. REMS design is a particularly timely topic to consider in the context of the steady increase in the use of fast-track, breakthrough therapy, accelerated approval, and priority review programs for drugs for serious life-threatening conditions, especially in the development of drugs and biologics for rare diseases, which may have a more limited study population and safety data. Therefore, it is valuable to use a systematic approach, such as a logic model, to assess for differences between premarketing evidence on safety and efficacy and postmarketing context and real-world use. Moreover, generic drugs account for more than 90% of prescriptions filled in the USA [8], and generic entry presents unique operational challenges in implementing shared systems and comparable REMS across products and companies over a drug’s lifespan. Further, concerns have been raised about the overall effectiveness of REMS programs and the quality of data submitted by manufacturers to evaluate REMS implementation and health outcomes [8, 9].

To address these challenges and further advance the scientific approach to REMS programs, the FDA recently released the draft Guidance for Industry entitled *REMS Logic Model: A Framework to Link Program Design with Assessment* [10]*.* Figure 1 shows the REMS logic model, which is a visual representation of the relationships between a program’s goals, activities, and outcomes through the design, implementation, and evaluation phases of a program. Leveraging this type of systematic approach to guide program design could result in more purposefully designed programs that reduce inefficiencies and achieve better program and health outcomes. The guidance is intended to describe the logic model to better link REMS assessment with a priori design assumptions in much the same way a clinical trial protocol is logically structured to link hypotheses to underlying biomedical disease assumptions and to their assessment and the statistical analysis plan.

**Fig. 1:**
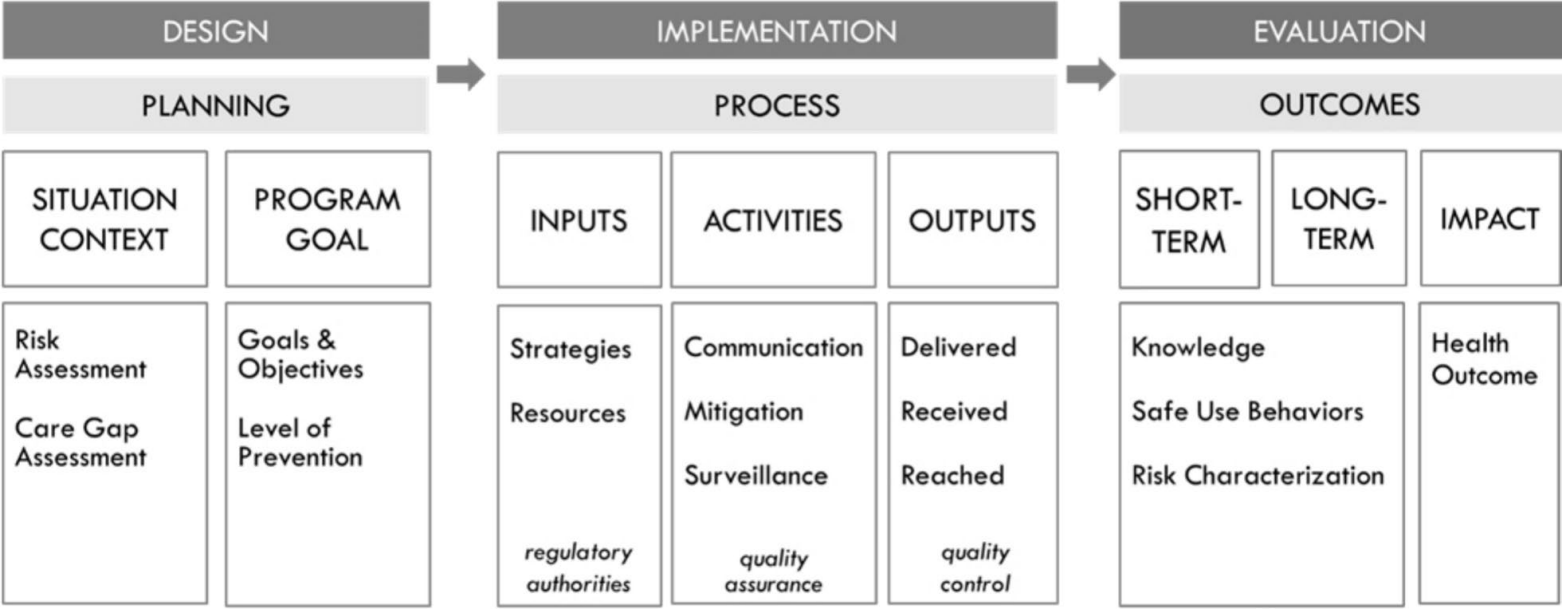
Risk evaluation and mitigation strategies (REMS) logic model

Implementation science and quality improvement behavior change frameworks, which include the use of logic models, have been widely adopted for clinical quality improvement intervention and public health program design, implementation, and evaluation, including pharmaceutical risk minimization programs [11–14]. In fact, recent revisions to the European Medicines Agency’s *Guideline on good pharmacovigilance practices (GVP) Module XVI—Risk minimisation measures* recognized that “Implementing RMM in healthcare for patient safety requires approaches from the implementation sciences as well as engagement across different stakeholders for patient-centered healthcare” [15].

Logic models are public health and healthcare quality improvement tools that help bridge the theory–research–practice divide [16, 17]. They have been used in program evaluation since the 1960s and in recent years have been increasingly applied for performance measurement and evaluation [18]. Logic models provide an intuitive clear and concise way of presenting the key elements of a program and how they relate to one other. By creating a visual representation of the relationships between program inputs, activities, outputs, and outcomes, the logic model makes explicit the scientific evidence, assumptions, and underlying reasoning that support the program’s design and the various processes behind it.

The systematic, structured approach of the FDA’s REMS logic model is designed to guide thinking and facilitate discussion to better link REMS program design, implementation, and evaluation. It makes transparent the evidence, assumptions, and uncertainties about the risk and risk mitigation measures and maps out what the REMS can and cannot accomplish. The model incorporates information on situational context to ensure that a program is designed to address specific care gap needs considering practical real-world practice constraints that affect patient access and healthcare system burden. The model can also be helpful to drug manufacturers and the agency to determine whether the program was implemented with fidelity. The model can help identify what is important to measure to determine whether the program is working as intended and achieving the desired public health outcomes. Finally, the model is flexible and adaptive and can be modified to improve program design and outcomes and inform REMS modifications.

The FDA recently applied the REMS logic model to re-evaluate the clozapine REMS and possible changes to minimize burden on patients, pharmacies, and prescribers while maintaining the safe use of clozapine [19, 20]. The “program goal” of the clozapine REMS is to mitigate the risk of severe neutropenia associated with the use of clozapine, an antipsychotic used for treatment-resistant schizophrenia and to reduce suicidal behavior in patients with schizophrenia or schizoaffective disorder. The program targets the secondary “level of prevention” (i.e., periodic screening of absolute neutrophil counts). The program implements safe-use behavior, education, and surveillance strategies through an integrated set of processes to ensure prescribers, patients, and pharmacies are enrolled so that these participants complete required “activities” to be trained and document and submit absolute neutrophil count results [19]. The FDA’s re-evaluation focused on the current “situation context” and whether the “risk assessment” and “care gap assessment” had changed since the clozapine risk management program was first implemented in 1989 [19]. Based on extensive data review and discussion at a November 2024 advisory committee meeting convened by the FDA, committee members were largely in agreement that the care gaps identified in 1989 had been addressed and a REMS was no longer necessary [21]. This example demonstrates how the REMS logic model can be applied throughout a product’s life cycle to continuously inform and validate the risk management design assumptions as new data become available, and the clozapine advisory committee meeting showed that structured discernment in action. Subsequently, the FDA determined that the clozapine REMS was no longer necessary to manage the drug’s risk of severe neutropenia and “eliminating the REMS is expected to decrease the burden on the health care delivery system and improve access to clozapine [22] .”

In summary, the FDA’s goal is to maintain patient access to effective medicines while preserving their safe use. The use of logic models is a helpful tool for bridging between premarketing evidence on safety and efficacy and postmarketing context and real-world use. The REMS logic model provides a roadmap to make more transparent and reproducible drug safety programs that are adaptive to an evolving, more digital healthcare system. Readers are encouraged to review the draft guidance in more detail for how the approach can be used in practice. The FDA is soliciting feedback on its draft guidance from all stakeholders to ensure that the agency considers public comment before it begins work on the final version of the guidance [10].

## References

[1] CDC National Center for Health Statistics. Therapeutic Drug Use. 2024 November 3, 2023 [cited 2024 July 7]. https://www.cdc.gov/nchs/fastats/drug-use-therapeutic.htm.

[2] Shehab N, Lovegrove MC, Geller AI, Rose KO, Weidle NJ, Budnitz DS. US emergency department visits for outpatient adverse drug events, 2013–2014. JAMA. 2016;316(20):2115–25. 27893129 10.1001/jama.2016.16201 PMC6490178

[3] 2007 US Food and Drug Administration Amendments Act (FDAAA). Section 505-1 of the FD&C Act applies to applications for prescription drugs submitted or approved under subsections 505(b) (ie, new drug applications) or (j) (ie, abbreviated new drug applications) (21 USC 355(b) or (j)) of the FD&C Act and to applications submitted or licensed under section 351 (ie, biologics license applications) of the Public Health Service Act (42 USC 262). U.S.; 2007.

[4] U.S. Food and Drug Administration. Risk evaluation and mitigation strategy (REMS) public dashboard. 2025 January 15, 2025 [cited 2025 January 20]. https://www.fda.gov/drugs/risk-evaluation-and-mitigation-strategies-rems/risk-evaluation-and-mitigation-strategy-rems-public-dashboard.

[5] U.S. Food and Drug Administration. Approved risk evaluation and mitigation strategies (REMS). 2024 [cited 2024 October 28]. https://www.accessdata.fda.gov/scripts/cder/rems/index.cfm.

[6] Toyserkani GA, Lee JH, Zhou EH. The risk evaluation and mitigation strategy (REMS) public dashboard: improving transparency of regulatory activities. Pharm Med. 2023;37(5):349–53. 10.1007/s40290-023-00489-5 37421560

[7] Association for Accessible Medicines. The U.S. Generic & Biosimilar Medicines Savings Report. 2023 [cited 2024 June 12]. https://accessiblemeds.org/sites/default/files/2023-09/AAM-2023-Generic-Biosimilar-Medicines-Savings-Report-web.pdf.

[8] Office of the Inspector General. FDA lacks comprehensive data to determine whether risk evaluation and mitigation strategies (REMS) improve drug safety. 2013 [cited 2024 June 1]. https://oig.hhs.gov/reports-and-publications/all-reports-and-publications/fda-lacks-comprehensive-data-to-determine-whether-risk-evaluation-and-mitigation-strategies-improve-drug-safety/.

[9] Office of the Inspector General. FDA's risk evaluation and mitigation strategies: uncertain effectiveness in addressing the opioid crisis. 2020 [cited 2024 June 1]. https://oig.hhs.gov/oei/reports/oei-01-17-00510.asp.

[10] U.S. Food and Drug Administration. REMS logic model: a framework to link program design with assessment guidance for industry DRAFT GUIDANCE. 2024 [cited 2024 June 1]. https://www.fda.gov/media/178291/download.

[11] Smith MY, Morrato E. Advancing the field of pharmaceutical risk minimization through application of implementation science best practices. Drug Saf. 2014;37(8):569–80. 25005707 10.1007/s40264-014-0197-0 PMC4134476

[12] Morrato EH. Social science theory as a framework for designing and evaluating pharmaceutical risk management dissemination and implementation strategies. In: Sprafka JM, editor. Risk management principles for devices and pharmaceuticals. 3rd ed. Rockville: Regulatory Affairs Professionals Society; 2023.

[13] Morrato EH, Smith MY. Chapter 13: dissemination and implementation science. In: Bahri P, editor. Communicating about risks and safe use of medicines: real life and applied research. Singapore: Springer Nature; 2020. p. 385–413.

[14] Bahri P. Public pharmacovigilance communication: a process calling for evidence-based, objective-driven strategies. Drug Saf. 2010;33(12):1065–79. 21077698 10.2165/11539040-000000000-00000

[15] Guideline on good pharmacovigilance practices (GVP) Module XVI—risk minimisation measures (Rev 3) 26 July 2024. 2024 [cited 2024 October 28]. https://www.ema.europa.eu/en/documents/regulatory-procedural-guideline/guideline-good-pharmacovigilance-practices-gvp-module-xvi-risk-minimisation-measures-rev-3_en.pdf.

[16] Kenyon CC, Palakshappa D, Feudtner C. Logic models-tools to bridge the theory-research-practice divide. JAMA Pediatr. 2015;169(9):801–2. 26214604 10.1001/jamapediatrics.2015.1365

[17] Foundation WKK. Using logic models to bring together planning, evaluation, and action: logic model development guide. 2004 January 2004 [cited 2024 October 28]. https://www.naccho.org/uploads/downloadable-resources/Programs/Public-Health-Infrastructure/KelloggLogicModelGuide_161122_162808.pdf.

[18] McLaughlin JA, Jordan GB. Chapter 3: “using logic models”. In: Joseph S. Wholey, Harry P. Hatry, Newcomer KE, editors. Handbook of practical program evaluation, 3rd ed. San Francisco: Jossey-Bass; 2010.

[19] U.S. Food and Drug Administration. FDA Briefing Document: Risk Evaluation and Mitigation Strategy (REMS) for Clozapine Products. Clozapine REMS Joint Meeting of the Drug Safety and Risk Management Advisory Committee and the Psychopharmacologic Drugs Advisory Committee November 19, 2024. 2024 [cited 2025 January 10]. https://www.fda.gov/media/183546/download.

[20] U.S. Food and Drug Administration. Risk Evaluation and Mitigation Strategy (REMS) for Clozapine Products—FDA Presentation Slides. 2024 [cited 2025 January 10]. https://www.fda.gov/media/183654/download.

[21] U.S. Food and Drug Administration. Summary Minutes of the Drug Safety and Risk Management Advisory Committee and Psychopharmacologic Drugs Advisory Committee Meeting November 19, 2024. December 12, 2024 [cited 2025 January 10]. https://www.fda.gov/media/184480/download#:~:text=Topic:%20The%20Committees%20discussed%20the%20reevaluation%20of,Administration%20were%20approved%20on%20Dec%2012%202024.

[22] U.S. Food and Drug Administration. Information on Clozapine. Updated February 25, 2025. https://www.fda.gov/drugs/postmarket-drug-safety-information-patients-and-providers/information-clozapine. Accessed March 18, 2025.

